# The Long Non-coding RNA LINC01705 Regulates the Development of Breast Cancer by Sponging miR-186-5p to Mediate TPR Expression as a Competitive Endogenous RNA

**DOI:** 10.3389/fgene.2020.00779

**Published:** 2020-07-31

**Authors:** Chuang Du, Jun-ling Zhang, Yan Wang, Ying-ying Zhang, Jian-hua Zhang, Lin-feng Zhang, Jing-ruo Li

**Affiliations:** ^1^Department of Breast Surgery, The First Affiliated Hospital of Zhengzhou University, Zhengzhou, China; ^2^Department of General Surgery, Peking University First Hospital, Beijing, China

**Keywords:** breast cancer, LINC01705, ceRNA, proliferation, cell invasion

## Abstract

Long non-coding RNAs (lncRNAs) may be a regulatory factor of tumorigenesis. However, it is unclear what its biomechanisms are in breast cancer. In this study, different lncRNAs were detected in breast cancer through microarray analysis (GSE119233) and LINC01705 was selected for further study. qRT-PCR was then utilized for the detection of LINC01705 expression in breast cancer cells. A transwell assay, flow cytometry, 5-ethynyl-2′-deoxyuridine (EdU), a cell counting Kit-8 (CCK-8), and a wound-healing assay were performed to determine cell migration, invasion, apoptosis, and proliferation in breast cancer, respectively. For the identification of potential targets of LINC01705, dual-luciferase reporter gene and bioinformatics assays were conducted. Moreover, for the clarification of their interaction and roles in the regulation of the occurrence of breast cancer, Western blotting and RIP assays were conducted. Our findings revealed high LINC01705 expression in breast cancer tissues relative to adjacent non-cancerous tissues (*n* = 40, *P* < 0.001). Overexpression of LINC01705 notably enhanced cell migration and proliferation in breast cancer. In addition, LINC01705 positively regulated the translocated promoter region, nuclear basket protein (TPR) through competition with miR-186-5p. In conclusion, our results suggest that LINC01705 is implicated in the progression of breast cancer via competitively binding to miR-186-5p as a competing endogenous RNA (ceRNA), thereby regulating TPR expression.

## Introduction

Breast cancer is not only the most prevalent malignant tumor in females, but is also one of the major causes of death from cancer in females around the world (Sarosiek, 2017). According to global cancer statistics, there were over 1.7 million newly diagnosed patients with breast cancer in 2012, taking up 25% of the incidence rate of all cancers (Woolston, 2015). Early stage breast cancer responds well to radiation, drug therapy, and surgical intervention (Fan et al., 2014). Nevertheless, in breast cancer there is a trend of metastasis to distant organs (e.g., brain, lungs, liver, and bones) and a poor prognosis is observed in patients with distant metastases. Despite advances in systemic chemotherapy, the median survival of patients with metastatic breast cancer is less than 2 years (DeSantis et al., 2011; Ganz and Goodwin, 2015). Therefore, in addition to existing breast cancer treatments such as surgical treatment, radiotherapy, chemotherapy, and immunotherapy, scientists need to explore more effective means of diagnosing breast cancer early, predicting its prognosis, inhibiting its metastasis, and reducing the mortality of breast cancer patients.

Long non-coding RNAs (lncRNAs) mean the non-coding RNAs with >200 nucleotides (Jarroux et al., 2017). Recent years, they have attracted much attention for their complex biological actions. Some lncRNAs have been found to be crucial in the migration, infiltration, invasion, proliferation, apoptosis and other cell functions of certain tumor cells (Wang M. W. et al., 2017; Wei and Wang, 2017; Zhang M. et al., 2017). For instance, the lncRNA DANCR enhances gastric cancer cell invasion and migration by suppressing lncRNA-LET (Mao et al., 2017). 1-IT1 represses colorectal cancer cell proliferation, metastasis, and invasion (Zhang W. et al., 2017). SNHG7 exerts enhancing effects on cell migration, proliferation, and invasion and inhibiting effects on lung cancer cell apoptosis by increasing FAIM2 expression (She et al., 2016). Most of lncRNAs exert its biological functions via functioning as ceRNA to regulate the expression of their target genes (He et al., 2018; Dong et al., 2019; Wang et al., 2019). Here, different lncRNAs were detected in breast cancer through microarray analysis (GSE119233). We found that LINC01705 is significant overexpression in breast cancer samples. Then we verified this result in breast cancer tissues and cell lines. Thus, LINC01705 was selected for further study. However, there has been scarcely any study of LINC01705.

Translocated promoter region, nuclear basket protein (TPR) is a prominent architectural component of the nuclear pore complex. TPR was originally described in the context of oncogenic fusions with the receptor tyrosine kinases Met, TRK, and Raf (Snow and Paschal, 2014). TPR has been since implicated in a variety of nuclear functions, including mitosis, regulation of transcription, chromatin organization, and nuclear transport (Snow and Paschal, 2014). A study showed that high expression of TPR is associated with ependymoma and TPR may act as a biomarker for ependymoma (Dewi et al., 2020). TPR is also related to some other cancer progression, including colorectal cancer (Dewi et al., 2018), lung adenocarcinoma (Choi et al., 2014) and so on.

In our study, the GSE119233 data was screened in order to identify the differential expression of lncRNAs in breast cancer and adjacent non-cancerous tissues, and tissues were further harvested for later quantitative real-time PCR (qRT-PCR). LINC01705 was utilized as the object of this study. Our results revealed overexpression of LINC01705 in breast cancer cells and tissues, and overexpression of LINC01705 promoted the proliferation and migration of BT-549 and MCF-7 cells. Collectively, this study confirmed that LINC01705 is implicated in the progress of breast cancer via sponging miR-186-5p, thereby mediating the expression of the TPR, providing a novel perspective for the study of the pathogenesis of breast cancer.

## Materials and Methods

### Editorial Policies and Ethical Considerations

The Ethics Committee of The First Affiliated Hospital of Zhengzhou University expressed their approval of this study.

### Patients and Specimens

Forty paired breast cancer tissues and adjacent non-cancerous tissues were collected from the breast cancer patients undergoing surgery. Then, the tissues were confirmed by pathology. Special forceps were used to harvest fresh tissues from cancerous lesions and adjacent normal tissues. A professional physician immediately washed the samples using DEPC, then placed them into a refrigerated tube. Then the refrigerated tubes were labeled and placed into a liquid nitrogen tank for freezing. The correlation between LINC01705 level and the clinicopathological parameters of forty breast cancer patients was shown in Supplementary Table 1.

### Cell Culture and Transfection

The human normal mammary epithelial cell line MCF 10A and the breast cancer cell lines CAL-51, MCF-7, BT-20, BT-549, and AU565 were commercially acquired from the Chinese Academy of Sciences Cell Bank (Shanghai, China). Cells were cultured in DMEM (Gibco, El Paso, TX, United States) containing 10% fetal bovine serum (FBS, Beyotime, Nantong, China), 100 IU/ml penicillin and 100 μg/ml streptomycin (Invitrogen, Carlsbad, CA, United States), and maintained at 37°C with 5% CO_2_.

Cell transfection was performed according to previous study (Zhu et al., 2019). LINC01705 cDNAs were amplified from human breast cancer tissues. Then, pLVX-LINC01705 vectors were constructed after the cDNAs were cloned into the *Bam*HI and *Xho*I sites of pLVX-IRES-Neo vectors (Invitrogen, Carlsbad, CA, United States). Subsequently, Lipofectamine 2000 (Invitrogen, Carlsbad, CA, United States) was used to transfect HEK293T cells with pLVX- LINC01705 vectors to package lentiviruses, which were used to infect breast cancer cells. MiR-186-5p mimics, miR-186-5p inhibitor, miR-NC, and inhibitor-NC were obtained from Genechem (Shanghai, China).

### RNA Extraction and qRT-PCR

After reverse transcription of RNAs into cDNAs using a Reverse Transcription Kit (Takara, Tokyo, Japan), RNA quantification was performed. GAPDH was used as an endogenous control for lncRNAs and mRNAs. The expression of miRNA was normalized to U6. The 2^–Δ^ ^Δ^ ^*CT*^ method was used to calculate relative expression levels. The primers’ sequences are shown in Table 1. All the experiments were independently conducted three times.

**TABLE 1:** Sequences of primers for qRT-PCR.

**Name**		**Sequence**
LINC01705	Forward	5′-TTCTGTCTTAGGGCATGGCA-3′
	Reverse	5′-AGAGCTGAGAGTTGGGGAAC-3′
TPR	Forward	5′-AACGCCAGCGTGAGGAATATG-3′
	Reverse	5′-ATTACGTGGTTACCCCTTGCT-3′
GAPDH	Forward	5′-GGTCACCAGGGCTGCTTTTA-3′
	Reverse	5′-GGATCTCGCTCCTGGAAGATG-3′
U6	Forward	5′-CTCGCTTCGGCAGCACA-3′
	Reverse	5′-AACGCTTCACGAATTTGCGT-3′
miR-186-5p	Forward	5′-ACACTCCAGCTGGGCAAAGAATTCTCCTTT-3′
	Reverse	5′-CTCAACTGGTGTCGTGGAGTCGGCAATTCAGT TGAGAGCCCAAA-3′

### Cell Proliferation Assay

After cell culture in 96-well plates, 1 h of incubation was performed using cell counting Kit-8 (CCK-8) reagent (Beyotime, Nantong, China). Then, a TECAN infinite M200 Multimode microplate reader (Tecan, Mechelen, Belgium) was employed to record the absorbance at 450 nm.

For a 5-ethynyl-2′-deoxyuridine (EdU) assay, 2 h of cell incubation was conducted in EdU reagent (Keygen, Nanjing, China). Before EdU staining following the manufacturer’s recommendations, 15 min of cell fixation was performed in 4% paraformaldehyde (Beyotime, Nantong, China).

### Wound-Healing Assay

Cell culture was completed until 80% of cell fusion in 6-well plates. Then, a 200-μL pipette tip was utilized for monolayer scratching. After rinsing using PBS and culture in FBS-free medium (Gibco, El Paso, TX, United States), a microscope was used to observe and photograph the wounds at 0 and 24 h. In addition, the wound size was measured with ImageJ (National Institutes of Health, Bethesda, MD, United States).

### Cell Invasion Determination

Cell invasion assay was performed according to previous study (Zhang et al., 2020). Transwell chambers (Millipore Corporation, Billerica, MA, United States) coated with Matrigel (BD Biosciences) were used to test cell invasion. The upper Transwell chamber was inoculated with the transfected cells (2.0 × 10^4^ per well) in DMEM without serum, whereas the lower Transwell chamber was filled with medium with 10% FBS. After incubating for 48 h at 37°C with 5% CO_2_, the cells not subjected to invasion in the upper chamber were rubbed away using a cotton swab, and those that underwent invasion into the lower chamber were subjected to 20 min of fixation in 4% paraformaldehyde, followed by 15 min of 1% crystal violet (Beyotime, Nantong, China) staining. Invasive cells were photographed and quantified in five fields of view randomly selected using an optical microscope.

### Cell Apoptosis and Cell Cycle Assays

Apoptotic cells were detected using an Annexin V-FITC/PI Apoptosis Detection Kit (Beyotime, China). Briefly, the cells were washed with PBS three times and stained with 5 μL Annexin V-FITC for 5 min in the dark at room temperature, followed by the addition of 10 μL Propidium Iodide (PI) for 15 min. Next, the samples were measured using BeamCyte (China) and analyzed with CytoSYS 1.0 software.

A Cell Cycle and Apoptosis Kit (Beyotime, China) was utilized to observe the cell cycle. Briefly, cells were washed with pre-cooled PBS and fixed with 70% ethanol for 24 h overnight. After that, 0.2% Triton X-100 was added to the cell solution, followed by resuspension with 100 μg/mL RNAase A for 30 min. Next, 10 μL PI solution was added for staining at 37°C in the dark. BeamCyte (China) was used to analyze the results.

### Subcellular Distribution

A PARIS Kit (Life Technologies, United States) was used for RNA extraction from the nucleus and cytoplasm. QRT-RCR was used for the quantification of Total RNA from each fraction. U6 and GAPDH were used as internal references for the nucleus and cytoplasm, respectively.

### Dual-Luciferase Reporter Gene Assay

This assay was performed according to previous study (Chen et al., 2020). Mutant-type plasmids LINC01705-MUT and TPR-MUT, and wild-type plasmids LINC01705-WT and TPR-WT were constructed. MCF-7 and BT-549 cells were seeded into 24-well plates. Subsequently, Lipofectamine 2000 was used for co-transfection with 50 nM miR-186-5p mimics or a negative control and mutant or wild-type plasmids, with 5 ng pRL-SV40 for each 80 ng plasmid. Luciferase intensity was determined on a microplate reader with a dual-luciferase reporter assay kit (Promega, Madison, WI, United States).

### RNA Immunoprecipitation (RIP)

This assay was performed according to previous study (Chen et al., 2020). The RIP assay was performed using the Magna Nuclear RIP^TM^ (Native) Nuclear RNA-Binding Protein Immunoprecipitation Kit (Millipore, Bedford, MA, United States). Cell lysis was conducted in a complete RIPA buffer with an RNase inhibitor and protease inhibitor cocktail. RIP buffer containing magnetic beads was used to incubate the cell extract post conjugation to IgG control or human anti-AGO2 antibody (Millipore). Protein digestion generated immunoprecipitated RNAs. Finally, qRT-PCR was used to quantify the purified RNAs.

### Western Blotting

Protein samples were subjected to extraction and quantification using BCA, separation using SDS-PAGE gel electrophoresis, and blocking using 5% skim milk. Subsequently, membranes were subjected to incubation with the primary antibody (rabbit anti-human IgG antibody against GAPDH and TPR) and a matched secondary antibody. Band exposure was developed via chemiluminescence.

### Construction of Tumor Models

Five-week BALB/c athymic nude mice from the National Laboratory Animal Center (Beijing, China) were subjected to 7 days of acclimation before the assay. Zhengzhou University approved all operations in all mice. For the construction of a breast cancer xenograft model, 4 × 10^6^ BT-549 cells stably transfected with sh-LINC01705 (*n* = 4) or sh-NC (*n* = 4) were seeded in the dorsal right flank of each mouse. Tumor tissues were harvested for subsequent research on day 22.

### Statistical Processing

Statistical analyses were applied using GraphPad Prism 6.0 and SPSS 20.0 software. Quantitative data was reported as the mean ± s.d. For normally distributed data with equal variance, the difference was evaluated by 2-tailed Student’s *T*-test (2-group comparisons) or ANOVA followed by the *post hoc* Bonferroni test (multigroup comparisons) as appropriate. For non-normally distributed data or data with unequal variances, the difference was evaluated by a non-parametric Mann–Whitney U test (2-group comparisons) or the Kruskal–Wallis test followed by the *post hoc* Bonferroni test (multigroup comparisons). Pearson correlative analysis was used to test correlation among LINC01705, miR-186-5p, and TPR. The log-rank test was adopted for the assessment of survival rates. A *P*-value of less than 0.05 was deemed the threshold for statistical significance.

## Results

### LINC01705 Expression in Breast Cancer Tissues and Cells

**FIGURE 1:**
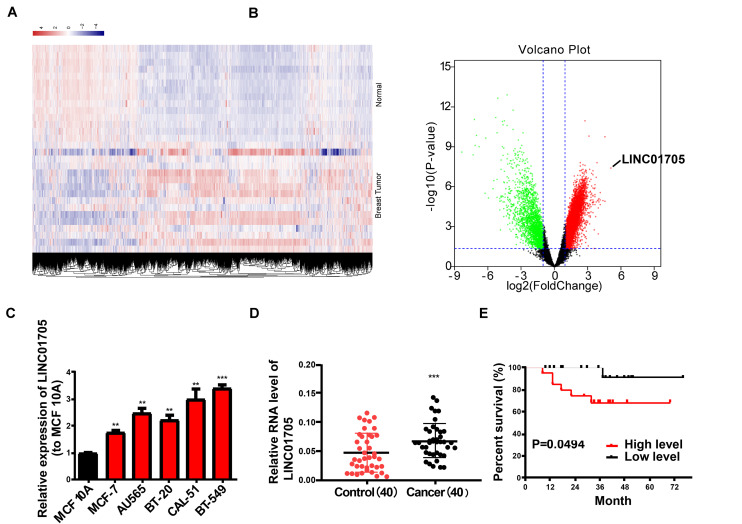
Features and expression of LINC01705 in breast cancer. **(A)** Heatmap and **(B)** volcano plot describing the dysregulation of 8602 lncRNAs, including the upregulation of 6131 lncRNAs, in breast cancer tissues (fold change >2 or <0.5, *P*-value < 0.05). **(C)** qRT-PCR of LINC01705 expression in breast cancer cell lines (MCF-7, BT-549, CAL-51, AU565, and BT-20) and the normal human mammary epithelial cell line MCF 10A. **(D)** qRT-PCR for the detection of LINC01705 expression in breast cancer tissues and para-carcinoma tissues. **(E)** A remarkably lower survival rate was observed in patients with high expression of LINC01705 than in patients with low expression of LINC01705. Data was reported as the mean ± s.d. **P < 0.01, ***P < 0.001.

To identify lncRNAs whose expression correlated with breast cancer, microarray analysis was employed to analyze the expression profiles of lncRNAs in 20 breast cancer tissues and 10 adjacent tissues (GSE119233). Collectively, a total of 8602 dysregulated lncRNAs (fold change <0.5 or >2, *P*-value < 0.05) were observed, including 6131 upregulated lncRNAs in breast cancer tissues (Figures 1A,B). Then, qRT-PCR was conducted to detect LINC01705 expression in breast cancer cells (MCF-7, CAL-51, BT-20, BT-549, and AU565) and human normal mammary epithelial cells MCF 10A; high expression of LINC01705 was observed in breast cancer cells (Figure 1C). Of the selected breast cancer cell lines, the highest and lowest levels of LINC01705 were observed in BT-549 cells and MCF-7 cells, respectively, which were selected for subsequent experiments. A prominent elevation of LINC01705 expression was identified in breast cancer tissues relative to adjacent tissues (Figure 1D). Forty breast cancer tissues were evenly stratified into a low-level group and a high-level group based on the median LINC01705 expression. The low-levlel group had a higher overall survival rate than the high-level group (Figure 1E). Then, we further explored the correlation between LINC01705 level and clinicopathological factors of breast cancer. Results showed that higher LINC01705 level was associated with TNM stage (Supplementary Table 1, *P* < 0.05).

### Roles of LINC01705 in Breast Cancer Cells

**FIGURE 2:**
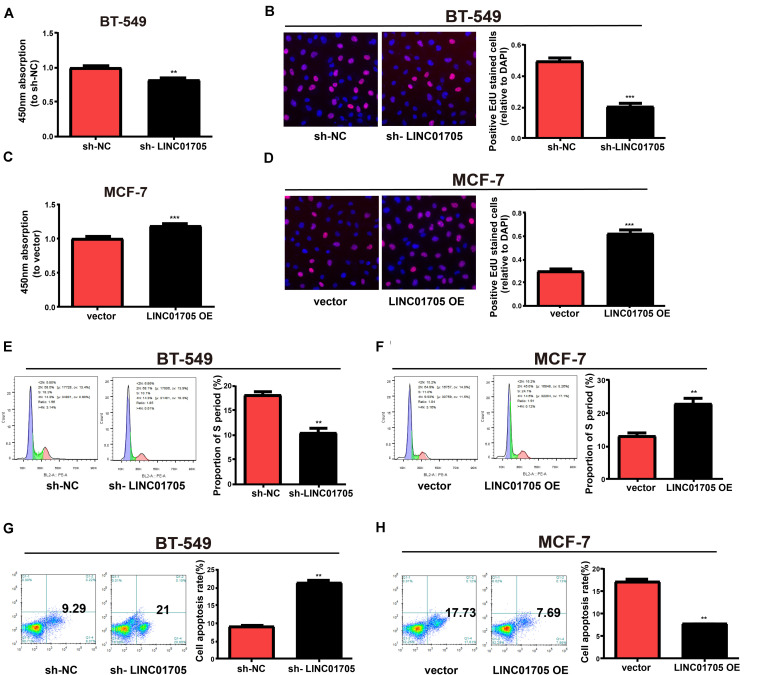
Regulatory effects of LINC01705 on proliferation, apoptosis, and cell cycle progression in breast cancer cells. **(A,B)** CCK-8 assay of BT-549 proliferation post-transfection with sh-LINC01705, and of MCF-7 cells post-transfection with LINC01705 OE. **(C,D)** EdU assay of BT-549 proliferation post-transfection with sh-LINC01705, and of MCF-7 cells post-transfection with LINC01705 OE. BD Biosciences FACS Calibur Flow Cytometry for **(E,F)** cell cycle and **(G,H)** cell apoptosis. Data was reported as the mean ± s.d. **P < 0.01, ***P < 0.001.

qRT-PCR was used to verify the transfection efficacy of the sh-LINC01705 and LINC01705 overexpression vectors in breast cancer cells (Supplementary Figures 1A,B). The proliferation of breast cancer cells was notably reduced by downregulation of LINC01705 (Figure 2A) and enhanced by overexpression of LINC01705 (Figure 2B), as shown by the CCK-8 assay. The EdU experiment generated the same results as the CCK-8 assay (Figures 2C,D). Cell cycle progression showed that the S phase in BT-549 cells with LINC01705 knockdown was shortened (Figure 2E). However, the S phase was extended in MCF-7 cells with LINC01705 overexpression (Figure 2F). The apoptosis experiment showed that the apoptosis rate was markedly increased in sh-LINC01705-transfected BT-549 cells (Figure 2G) and notably decreased in LINC01705 (overexpression) OE-transfected MCF-7 cells (Figure 2H).

**FIGURE 3:**
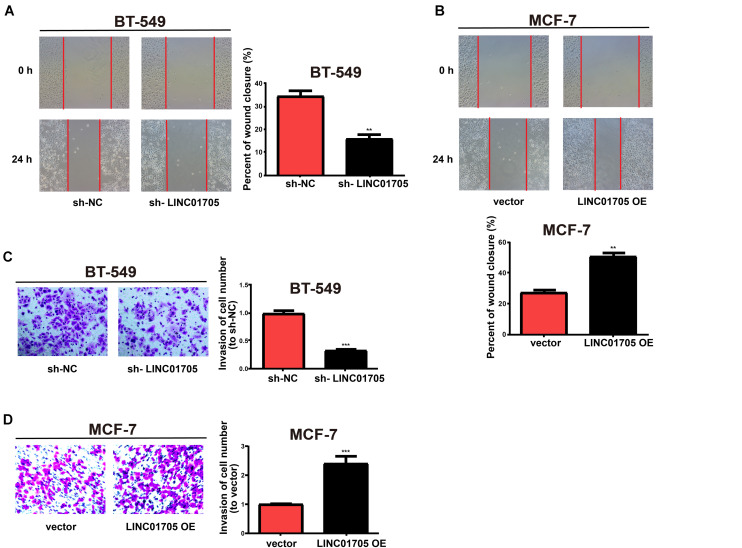
Regulatory effects of LINC01705 on migration and invasion of breast cancer cells. **(A,B)** Wound-healing assay for cell migration. **(C,D)** Transwell assay for BT-549 invasion post-transfection with sh-LINC01705, and for MCF-7 cells post-transfection with LINC01705 OE. Images were collected at ×200 using a light microscope. Data was reported as the mean ±s.d. **P < 0.01, ***P < 0.001.

In addition, based on the wound-healing assay, breast cancer cell migration was decreased by downregulation of LINC01705 (Figure 3A) and enhanced by overexpression of LINC01705 (Figure 3B). In addition, the invasion experiment showed that breast cancer cell invasion was decreased by downregulation of LINC01705 (Figure 3C) and enhanced by overexpression of LINC01705 (Figure 3D).

In summary, these results showed that overexpression of LINC01705 promoted cell migration, invasion, proliferation, and cell cycle progression in MCF-7 cells, but retarded cell apoptosis. Meanwhile, downregulation of LINC01705 inhibited cell migration, invasion, proliferation, and cell cycle progression in BT-549 cells, but promoted cell apoptosis.

### Subcellular Distribution of LINC01705

**FIGURE 4:**
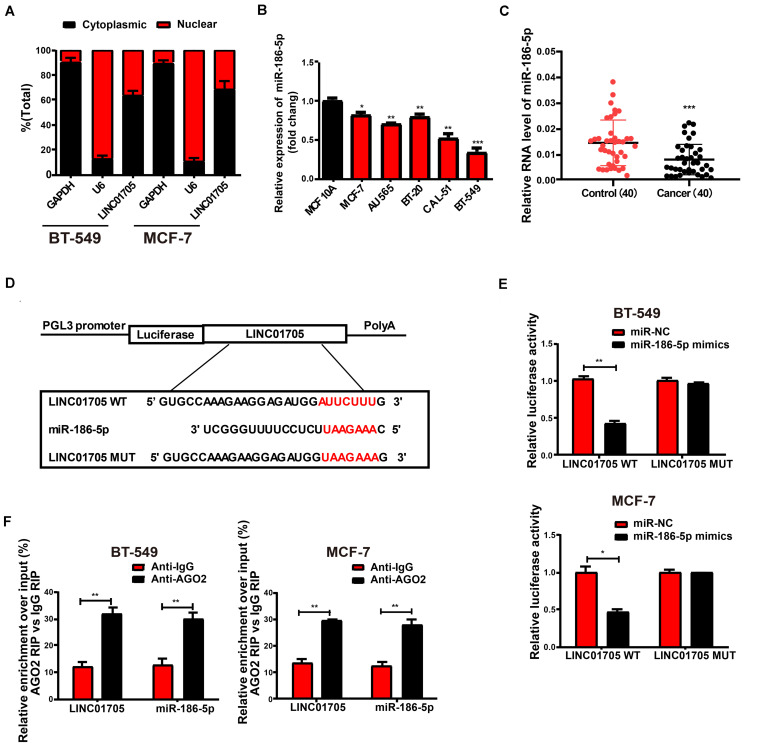
LINC01705 directly interacts with miR-186-5p. **(A)** qRT-PCR for nuclear and cytoplasmic levels of LINC01705 in BT-549 and MCF-7 cells. **(B)** qRT-PCR for miR-186-5p expression in breast cancer cell lines and a human normal mammary epithelial cell line. **(C)** qRT-PCR for MiR-186-5p expression in breast cancer tissues and adjacent tissues. **(D)** Bioinformatic evidence of binding of miR-186-5p to the 3′-UTR of LINC01705. **(E)** Dual-luciferase reporter gene assay for BT-549 and MCF-7 cells transfected with miR-NC or miR-186-5p mimics, renilla luciferase vector pRL-SV40, and reporter constructs. **(F)** RIP experiment to quantify LINC01705 and miR-186-5p in BT-549 and MCF-7 cells. Data was reported as the mean ± s.d. *P < 0.05, **P < 0.01, ***P < 0.001.

Biological actions are determined by the subcellular distribution of lncRNA (Chen, 2016). To confirm the cellular location of LINC01705, breast cancer cells were isolated into nuclear and cytoplasmic fractions and U6 and GAPDH were used as controls, respectively. qRT-PCR revealed the presence of LINC01705 in the cytoplasmic fractions of BT-549 and MCF-7 cells, respectively (Figure 4A). This suggests that LINC01705 is involved in the progression of breast cancer via post-transcriptional regulation.

### LINC01705 Is Targeted by miR-186-5p

As LINC01705 is mainly identified in cytoplasmic fractions, it was postulated that LINC01705 might exert its biological functions at post-transcription level (Su et al., 2018; Zhang et al., 2019). The RegRNA, Starbase prediction observed a close match of miR-186-5p to the LINC01705 3′UTR. Using qRT-PCR, we found that miR-186-5p expression was lower in breast cancer cells (Figure 4B), and was also lower than in para-carcinoma tissues (Figure 4C). The binding sequences in miR-186-5p that correspond to LINC01705 3′UTR were used as the basis for the construction of pGL3-LINC01705-WT and pGL3-LINC01705-MUT (Figure 4D). After co-transfection with LINC01705 WT and miR-186-5p mimics, BT-549 and MCF-7 cells exhibited a notable decrease in luciferase activity, which remained unchanged after co-transfection with LINC01705 MUT and miR-186-5p mimics (Figure 4E). RIP analysis elucidated whether LINC01705 participated in the RNA-containing ribonucleoprotein complex. qRT-PCR revealed enriched LINC01705 in anti-AGO2 antibody relative to controls. In addition, miR-186-5p produced similar results (Figure 4F). We ultimately found that miR-186-5p bound to LINC01705 *in vitro*.

### LINC01705 Regulates TPR, the Target Gene of miR-186-5p

**FIGURE 5:**
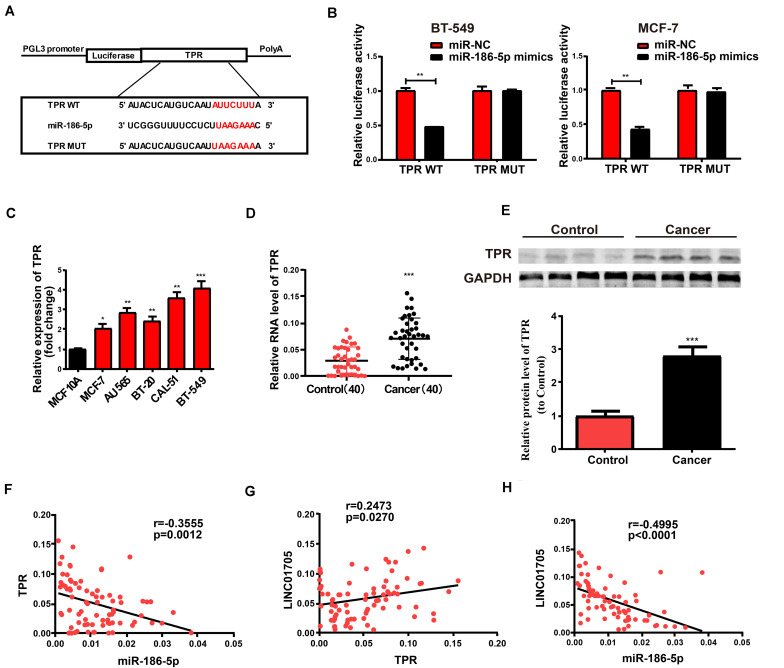
TPR is the direct target of miR-186-5p. **(A)** Hypothetic miRNA binding sites in the TPR sequence. **(B)** Confirmation of direct target sites by dual-luciferase reporter gene assay. **(C)** qRT-PCR for TPR expression in breast cancer cell lines and the human normal mammary epithelial cell line MCF 10A. **(D)** qRT-PCR for TPR expression in breast cancer tissues and adjacent tissues. **(E)** Western blotting for protein levels of TPR in breast cancer tissues and para-carcinoma tissues. **(F)** Inverse relationship between TPR in breast cancer tissues and miR-186-5p expression. **(G)** Positive correlation with LINC01705. **(H)** Negative correlation of miR-186-5p expression with LINC01705. Data was reported as the mean ± s.d. *P < 0.05, **P < 0.01, ***P < 0.001.

Bioinformatics prediction (TargetScan, Starbase, RegRNA) was employed to screen for the target gene of miR-186-5p in order to explore the role of miR-186-5p in the occurrence of breast cancer. TPR was selected for subsequent analyses. Constructed luciferase plasmids pGL3-TPR-WT and pGL3-TPR-MUT (Figure 5A) were subject to co-transfection with miR-186-5p mimics or miR-NC in BT-549 and MCF-7 cells, respectively. The WT reporter group showed suppressed luciferase activity while the MUT reporter group exhibited unchanged activity (Figure 5B). Such findings revealed that TPR was a possible target of miR-186-5p. Thereafter, qRT-PCR was utilized to determine TPR expression in breast cancer cell lines and tissues where the mRNA levels of TPR were remarkably elevated (Figures 5C,D). Meanwhile, similar results were observed in Western blotting analysis for protein levels (Figure 5E). Additionally, correlation analysis indicated that TPR expression in breast cancer tissues had a negative correlation with miR-186-5p expression and a positive correlation with LINC01705 expression (Figures 5F,G). In addition, LINC01705 expression was also negatively correlated with miR-186-5p expression (Figure 5H).

### LINC01705 Regulates TPR Expression by Targeting miR-186-5p

**FIGURE 6:**
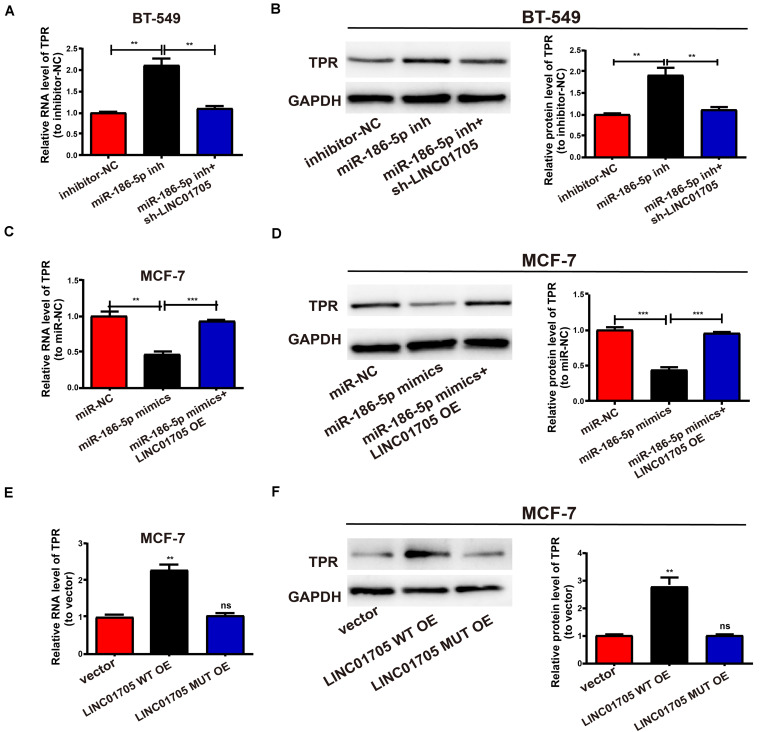
The LINC01705/miR-186-5p axis is critical for the expression of TPR. **(A,B)** After transfection of MiR-186-5p inhibitor into BT-549 cells with or without sh-LINC01705, qRT-PCR and Western blotting analysis were conducted to determine the mRNA and protein levels of TPR. **(C,D)** After transfection of MCF-7 cells with miR-186-5p with or without LINC01705 OE, qRT-PCR and Western blotting analysis were conducted to detect the mRNA and protein levels of TPR relative to controls. **(E)** Relative mRNA levels of TPR post transfection with LINC01705-WT overexpression plasmid or LINC01705-MUT overexpression plasmid. **(F)** Relative protein levels of TPR post transfection with LINC01705-WT overexpression plasmid or LINC01705-MUT overexpression plasmid. Data was reported as the mean ± s.d. **P < 0.01, ***P < 0.001, ns, no significant difference.

We next detected changes in TPR expression in breast cancer cells after altering LINC01705 or miR-186-5p expression to explore whether TPR was regulated by LINC01705 through targeting miR-186-5p. First, the transfection efficacy of miR-186-5p mimics and miR-186-5p inhibitor were verified in breast cancer cells by qRT-PCR (Supplementary Figures 1C,D). Upregulated TPR expression was observed post-transfection with miR-186-5p inhibitor in BT-549 cells, while co-transfection with miR-186-5p inhibitor and sh-LINC01705 had the reverse effect (Figures 6A,B). Moreover, TPR expression was suppressed post-transfection with miR-186-5p mimics in MCF-7 cells, while co-transfection with miR-186-5p mimics and LINC01705 overexpression plasmids had the reverse effect (Figures 6C,D). Before determining TPR expression, MCF-7 cells were subjected to transfection with LINC01705 WT OE and the corresponding mutant overexpression plasmid. Overexpression of wild-type LINC01705 enhanced TPR expression in breast cancer cells, as shown using both qRT-PCR and Western blotting. However, the LINC01705 to miR-186-5p base pairing was not disrupted by mutating LINC01705 (Figures 6E,F). Collectively, these findings verified that LINC01705 has a positive regulatory effect on TPR expression through direct binding to miR-186-5p.

### The LINC01705/miR-186-5p Axis Regulates the Behavior of Breast Cancer Cells

**FIGURE 7:**
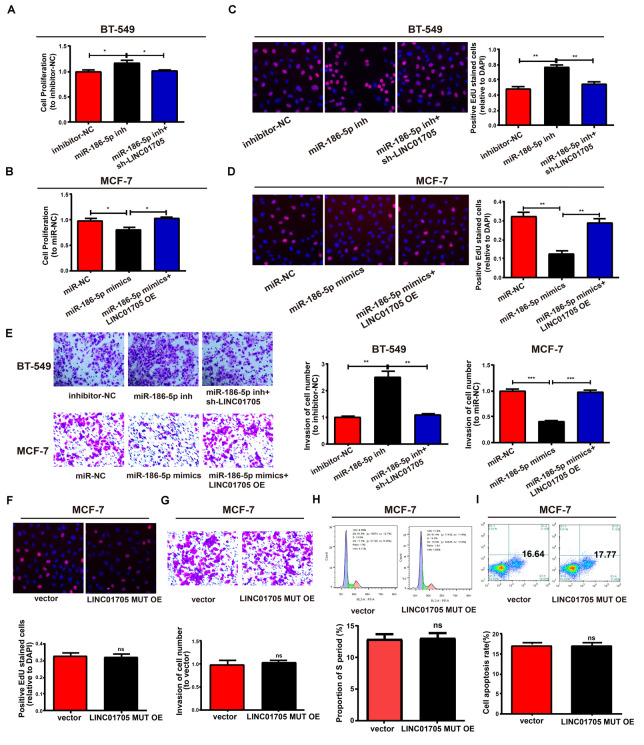
LINC01705 regulates cell function via miR-186-5p. **(A,B)** CCK-8 assay to determine the proliferation of BT-549 and MCF-7 cells. **(C,D)** EdU assay to measure the proliferation of BT-549 and MCF-7 cells. **(E)** Invasion of BT-549 and MCF-7 cell lines after altering transfections. **(F)** Detection of the proliferation, **(G)** invasion, **(H)** cell cycle progression, and **(I)** cell apoptosis after treatment of MCF-7 cells using the LINC01705-MUT overexpression plasmid. Data was reported as the mean ± s.d. *P < 0.05, **P < 0.01, ***P < 0.001, ns, no significant difference.

This study also clarified whether miR-186-5p could affect the proliferation and invasion of BT-549 and MCF-7 cells. Co-transfection with miR-186-5p inhibitor and sh-LINC01705 had a partial reversal effect on the notable enhancement of cell proliferation and invasion caused by downregulation of miR-186-5p relative to inhibitor-NC in BT-549 cells (Figures 7A,C,E). Furthermore, LINC01705 overexpression had a partial reversal effect on the proliferation and invasion changes in MCF-7 cells caused by overexpressed miR-186-5p (Figures 7B,D,E). Overexpression of mutant LINC01705 in MCF-7 cells did not affect the cell proliferation, invasion, cycle progression, or apoptosis (Figures 7F–I). By virtue of the above results, the LINC01705/miR-186-5p/TPR axis was shown to have a great influence on the behavior regulation of BT-549 and MCF-7 cells.

### The Effects of Knockdown of LINC01705 on Tumor Growth *in vivo*

**FIGURE 8:**
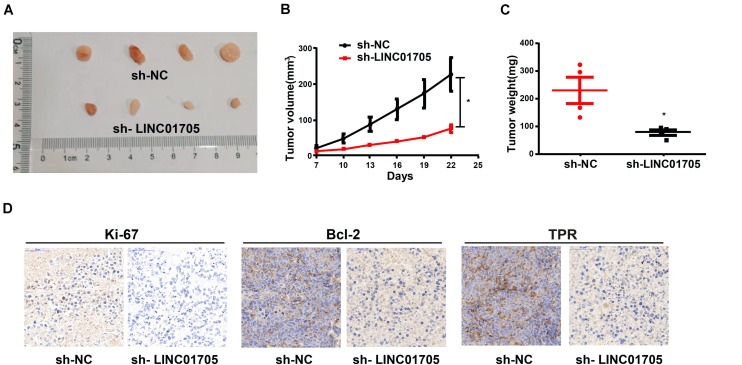
Sh-LINC01705 inhibits breast cancer growth *in vivo*. **(A)** Xenograft tumor. **(B)** The tumor growth of mice in each group was detected after BT-549 cells were inoculated subcutaneously. **(C)** Weight of Xenograft tumor. **(D)** Immunohistochemistry images after transfection. Data was reported as the mean ± s.d. *P < 0.05.

The verification of the role of LINC01705 in tumor growth was performed in nude mice, and revealed a lower tumor volume and weight in the knockdown LINC01705 group relative to the control group (Figures 8A–C). Also, the expression levels of Ki-67, Bcl-2, and TPR were reduced in the knockdown LINC01705 group (Figure 8D).

## Discussion

In spite of dramatic advances in cancer research, breast cancer remains a major health problem and currently represents one of the biomedical research priorities (Anastasiadi et al., 2017; Kolak et al., 2017). Cancer treatments are now decided based on underlying biomechanisms rather than the anatomic extent of the disease as before. LncRNAs have been reported to be a regulatory factor in many cellular processes (Quinn and Chang, 2016), and lncRNA dysregulation has been associated with the development of various diseases (Batista and Chang, 2013; Beermann et al., 2016) such as Parkinson’s disease (Majidinia et al., 2016), pancreatic cancer (Duguang et al., 2017), cervical cancer (Huang et al., 2017), and Alzheimer’s disease (Wan et al., 2017). Meanwhile, it has been previously observed that many lncRNAs participate in the occurrence and progression of breast cancer, including LncRNA-ATB (Shi et al., 2015), DSCAM-AS1 (Niknafs et al., 2016), and LncRNA BCAR4 (Xing et al., 2015). Microarray analysis has unveiled elevated LINC01705 expression in breast cancer. This, along with the lack of previous studies on LINC01705, makes our study highly significant.

This study revealed elevated LINC01705 expression in breast cancer cells and tissues. Moreover, downregulation of LINC01705 decreased cell cycle progression, proliferation, invasion, and migration in breast cancer, indicating that LINC01705 is a key regulatory factor in breast cancer cell growth as a carcinogene. Therefore, it has important sense to explore the influences of LINC01705 on the acceleration of breast cancer cell growth in order to further understand breast cancer occurrence, development, and metastasis.

The cytoplasm contains the main distribution of LINC01705, as confirmed by separating cytoplasmic and nuclear fractions, suggesting that LINC01705 may act as a ceRNA. LINC01705 binds to miR-186-5p, as shown via RIP and dual-luciferase reporter gene assays. Up until now, low miR-186-5p expression has been observed in non-small cell lung cancer (Wang H. et al., 2017), glioblastoma multiforme (Wang F. et al., 2017), and gastric cancer (Ouyang et al., 2019). Our study also revealed downregulated miR-186-5p expression in breast cancer cells and tissues. LINC01705 overexpression reversed the suppressive effects of transfection with miR-186-5p mimics on the invasion and proliferation of breast cancer cells. Thus, both LINC01705 and miR-186-5p are implicated in the development and progression of breast cancer.

Yang et al. (2018) found that the transcription of TPR was regulated by YAP, and that it was coordinately expressed with RACGAP1 to promote the proliferation of HCC cells by promoting cytokinesis. Wan et al. (2014) found that Tpr plays an important role in mitosis by stabilizing Mitotic arrest both deficient 1 (Mad1) and Mad2. Meanwhile, Mad1 overexpression accelerates directed cell migration. Our study also revealed TPR overexpression in breast cancer cells and tissues and its expression level is closely related to cell proliferation and migration of BT-549 and MCF-7 cells.

In summary, LINC01705 acts as a competitive endogenous RNA to regulate TPR expression via sponging miR-186-5p, thereby regulating the progression of breast cancer.

## Data Availability Statement

The raw data supporting the conclusions of this article will be made available by the authors, without undue reservation.

## Ethics Statement

The studies involving human participants were reviewed and approved by the Ethics Committee of The First Affiliated Hospital of Zhengzhou University expressed their approval of this study. The patients/participants provided their written informed consent to participate in this study. The animal study was reviewed and approved by the Ethics Committee of The First Affiliated Hospital of Zhengzhou University expressed their approval of this study.

## Author Contributions

JL designed the study. CD, JLZ, and YW performed the experiments and analyzed the data. CD was a major contributor in developing the first draft of this manuscript. YZ, JHZ, and LZ revised this manuscript. All authors read and approved the final version of the manuscript.

## Conflict of Interest

The authors declare that the research was conducted in the absence of any commercial or financial relationships that could be construed as a potential conflict of interest.
