# Supplementary material for: The Long Non-coding RNA LINC01705 Regulates the Development of Breast Cancer by Sponging miR-186-5p to Mediate TPR Expression as a Competitive Endogenous RNA
Source: Front Genet. 2020 Jul 31;11:779. doi: 10.3389/fgene.2020.00779 (PMC7412980; doi:10.3389/fgene.2020.00779)
Supplement: Supplementary file 1 [file Table_1.DOCX]

**Table 1. Patient Clinicopathologic features.**

| Clinicopathologic features | Number of cases | LINC01705 expression | | *P* value |
| --- | --- | --- | --- | --- |
|  |  | High  (n=20) | Low  (n=20) |  |
| Age |  |  |  | 0.7512 |
| <50 | 22 | 10 | 12 |  |
| ≥50 | 18 | 10 | 8 |  |
| ER status |  |  |  | 0.5273 |
| Negative | 19 | 11 | 8 |  |
| Positive | 21 | 9 | 12 |  |
| HER-2 status |  |  |  | 0.7311 |
| Negative | 28 | 15 | 13 |  |
| Positive | 12 | 5 | 7 |  |
| PR status |  |  |  | 1.0000 |
| Negative | 17 | 9 | 8 |  |
| Positive | 23 | 11 | 12 |  |
| TNM stage |  |  |  | **0.0248** |
| I/II | 18 | 5 | 13 |  |
| III | 22 | 15 | 7 |  |

Total data from 40 tumor tissues of breast cancer patients were analyzed. For the expression of LINC01705 was assayed by qRT-PCR, the median expression level was used as the cutoff. Data were analyzed by Fisher’s exact test. P-value in bold indicates statistically significant.
