# Supplementary material for: The Long Non-coding RNA LINC01705 Regulates the Development of Breast Cancer by Sponging miR-186-5p to Mediate TPR Expression as a Competitive Endogenous RNA
Source: Front Genet. 2020 Jul 31;11:779. doi: 10.3389/fgene.2020.00779 (PMC7412980; doi:10.3389/fgene.2020.00779)
Supplement: Supplementary file 2 [file Data_Sheet_1.DOCX]

**Supplementary Figure 1**

**
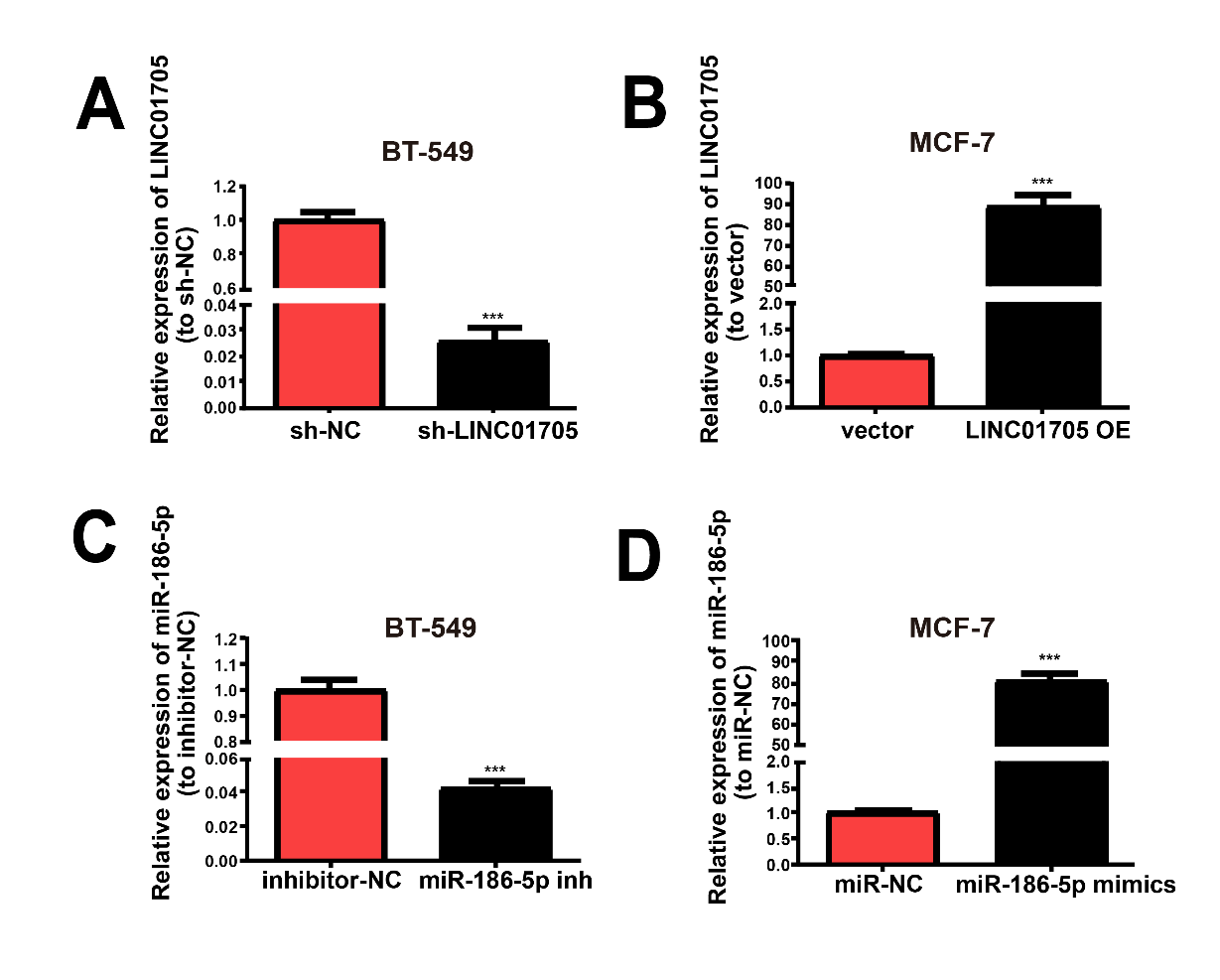
**

**Supplementary Figure 1.** (A) qRT-PCR was used to identify the effect of sh-LINC01705 in BT-549 cells. (B) The RNA level of LINC01705 was detected by qRT-PCR after treat with LINC01705 over-expressing vector. (C) qRT-PCR was used to identify the effect of miR-186-5p inhibitor in BT-549 cells. (D) The RNA level of miR-186-5p was detected by qRT-PCR after treat with miR-186-5p mimics in MCF-7 cells. ^***^*P*<0.001.
